# Prevalence and Risk Factors of Poor Sleep Quality among Chinese Elderly in an Urban Community: Results from the Shanghai Aging Study

**DOI:** 10.1371/journal.pone.0081261

**Published:** 2013-11-25

**Authors:** Jianfeng Luo, Guoxing Zhu, Qianhua Zhao, Qihao Guo, Haijiao Meng, Zhen Hong, Ding Ding

**Affiliations:** 1 Department of Health Statistics and Social Medicine, School of Public Health, Fudan University, The Key Laboratory of Public Health Safety of Ministry of Education, Shanghai, China; 2 Institute of Neurology, Huashan Hospital, Fudan University, WHO Collaborating Center for Research and Training in Neurosciences, Shanghai, China; Institute of Automation, Chinese Academy of Sciences, China

## Abstract

**Background:**

Sleep disorders causes a significant negative effect on mental and physical health, particularly among the elderly. The disease burden and risk factors of poor sleep quality of the elderly need to be verified using a validated form of measurement in urban mainland China.

**Methods:**

This study included 1086 community residents aged ≥60 years who completed the Chinese version of the Pittsburgh Sleep Quality Index (CPSQI). Poor sleeper was defined by a CPSQI global score of >5. Subjects also accepted the neurological and neuropsychological assessments, including the Mini-Mental State Examination, Center for Epidemiological Studies Depression Scale, and Zung Self-Rating Anxiety Scale (ZSAS). A history of chronic diseases was confirmed by the medical records of each participant.

**Results:**

The prevalence of poor sleep quality in this population was 41.5% (95% confidence interval (CI) = 38.6–44.5%), with a higher rate observed in elderly females (45.8% [95% CI = 41.9–49.7%]) than that in elderly males (35.8% [95% CI = 31.4–40.1%]). The prevalence rate increased with age, from 32.1% (95% CI = 27.8–36.4%) in those aged 60–69 years to 52.5% (95% CI = 45.9–59.1%) in those aged ≥80 years (p value for trend<0.001). Multivariate logistic regression analysis indicated that age (OR = 1.03[95% CI = 1.01–1.05], p<0.001), less education duration (OR = 1.04 [95% CI = 1.01–1.08, p = 0.014), living alone (OR = 1.62 [95% CI = 1.02–2.58], p = 0.04), anxiety (ZSAS score: OR = 1.09 [95% CI = 1.05–1.12], p<0.001), number of chronic disease (OR = 1.18 [95% CI = 1.07–1.30], p = 0.14) and arthritis (OR = 1.45[95% CI = 1.05–2.01], p = 0.025) were risk factors of poor sleep quality.

**Conclusions:**

Poor sleep quality is highly prevalent among elderly Chinese residents in urban Shanghai. Growing attention and comprehensive countermeasures involving psycho-social and personal activities might alleviate the sleep problem in the elderly.

## Introduction

Sleep disorder is a common complaint among the general populations of western countries, with rates of self-reported insomnia ranging between 10% and 48% [1]–[4]. Sleep disorder has a significantly negative impact on morbidity and mortality, particularly in the elderly population [5]–[8]. Inability to sleep can lead to difficulty sustaining attention, a slowed response time, impairments in memory and concentration, and decreased performance. These symptoms are of particular concern in older people, because they may be misinterpreted as symptoms of dementia or mild cognitive impairment [9], [10]. Slowed response time is particularly important because it can affect driving ability and increase the risk of falls [11]. Perhaps most startling is that insomnia is also associated with shorter survival. Mortality due to common causes of death (e.g., heart disease, stroke, cancer, suicide) is up to two times higher in elderly with sleep disorders than in those who sleep well [12], [13].

Given the rapidly ongoing aging of the population, health care professionals and multidisciplinary researchers have recently begun to pay closer attention to the sleep quality among the elderly in low-income countries. Several global studies indicated that a large number of elderly in low-income settings are currently experiencing sleep problems which linked to poorer general well-being, quality of life, and psychiatric comorbidities [14]–[16]. There are about 0.18 billion elderly older than 60 years in China, accounting for 13.3% of whole population according to the 6th Chinese national census. The elderly over 60 years was estimated to reach 0.48 billion in 2050. Several population-based studies in China have demonstrated that the prevalence of sleep disorder among elderly varied from 6% to 40% [16]–[24]. However, it's difficult to compare these studies due to the substantial variety of diagnostic definitions, detection procedures, implementation instruments, and demographic characteristics of source population.

Sleep quality is an important clinical construct and essential part of quality of life. It includes quantitative aspects of sleep, eg. sleep duration, sleep latency, or number of arousals as well as more pure subjective aspects, such as "depth" or "restfulness" of sleep [25]. Most previous studies in China only used simple unstructured questions like “how many hours on average do you sleep every day”, “have you had trouble sleeping recently”, or “how do you rate your sleep quality recently”. These questions may be insufficient to deliver valid assessment of sleep quality. The Pittsburgh Sleep Quality Index (PSQI) was designed to evaluate sleep quality through 19 items on multiple dimensions of sleep over a 1-month period [25], [26]. The Chinese version of Pittsburgh Sleep Quality Index (CPSQI) has been validated. It can be a sensitive, reliable, and valid outcome assessment tool for use in population-based studies of primary insomnia [27]. It has been used in several studies in Taiwan and Hongkong Chinese, and resulted the prevalence of poor sleep quality ranging from 6% to 78% [21], [22], [28]. In mainland China, there was only one epidemiological study using CPSQI, reporting that 50% of elderly aged 60 or older had poor sleep quality in a sample population in rural area [21]. Data in urban China is still lacking. In this study, we aimed to fill this knowledge gap.

To reduce personal suffering and the harmful consequences caused by poor sleep quality, it is essential to understand the socio-demographic and clinical correlates of sleep disorder. It is regarded that aging, circadian rhythm disturbances, medical/psychiatric illnesses, drugs/medication, psychosocial factors have impact in the development, persistence, and treatment of sleep disorder in adults [5], [29], [30]. Besides female gender, enhanced age, divorced or separated marital status and low educational level, chronic diseases and abnormal somatic or psychiatric conditions were also reported to be associated with sleep disturbance in both western [5], [7] and eastern countries [19], [21], [22], [28]. However, most previous studies in the Chinese population only used invalidated self-reported information on the patient's overall subjective health status, and did not include any physician-diagnosed clinical and mental disorders.

In this population-based epidemiological study, we used the CPSQI to evaluate the subjective sleep quality in the elderly Chinese population living in an urban community, and to demonstrate the related risk factors of poor sleep quality in this population.

## Methods

### Ethics Statement

This study was approved by the Medical Ethics Committee of Huashan Hospital, Fudan University, Shanghai, China. A written informed consent was obtained from all of the participants and/or their legal guardian.

### Study site and subjects recruitment

Subjects who participated in the Shanghai Aging Study were recruited consecutively. The Shanghai Aging Study aimed to investigate the prevalence of dementia and mild cognitive impairment among elderly residing in an urban community of Shanghai. The inclusion criteria were that the participant was required 1) to be a registered resident of the Jingansi community, 2) to be age ≥60 years and 3) to provide a written informed consent for study participation. Subjects were excluded if they 1) were deceased; 2) demonstrated severe schizophrenia or mental retardation on their medical record; or 3) had severe problems of vision, hearing, or speaking, and were not able to participate.

Potential subjects were identified using a government maintained ‘residents list’, which included the name, sex, age, address, and telephone number of every resident. Study coordinators went to each home in each neighborhood to introduce the study information. People who were willing to participate and who met the inclusion criteria were consecutively enrolled. A clinical interview (either at Huashan Hospital, or at subjects' homes) appointment then was made. Participants were reminded of the evaluation by a telephone call one day before it was scheduled. In the first part of the Shanghai Aging Study, 1763 registered long-term residents were enrolled from four neighborhoods of the community. Among these subjects, 212 were ineligible and 465 did not wish to participate. Thus, 1086 subjects participated in the current study and had finished with the clinical interviews on sleep disorders.

### Neurological and neuropsychological assessments

The participants were interviewed face-to-face by trained neurologists to elicit information on the subject's demographic characteristics, including the patient's birth date, gender, and education. We measured the height and weight of each participant. The body mass index (BMI) was calculated as a person's weight in kilograms (kg) divided by their height in meters (m) squared. Other lifestyle factors, such as living situation, exercise regime, and smoking and drinking habits were obtained. A current smoker was defined as a person who had smoked daily within the past month. A current drinker has had at least one episode of alcohol drinking weekly during the past year. We asked the participants about their medical histories, as defined by physician-diagnosed hypertension, diabetes, and heart disease (including coronary artery disease and arrhythmia) and confirmed these medical histories from the participants' medical records.

To ensuring the consistence of neuropsychological measurements, two trained psychometrists were assigned to administer a Chinese Mini-Mental State examination (MMSE) [31], Center for Epidemiologic Studies Depression Scale (CESD) [32] and Zung Self-Rating Anxiety Scale (ZSAS) for each participant [33], which were validated in Chinese population. In this study, these exams were used to determine the patient's cognitive function and to assess whether each participant met the criteria of having a major depressive or anxiety episode within the past week.

### Measurement of sleep quality

PSQI is a self-rated questionnaire which assesses sleep quality and disturbances over a 1-month time interval. The CPSQI had a good overall reliability (r  =  0.82–0.83) and test-retest reliability (r  =  0.77–0.85) in community-dwelling adults with primary insomnia [27] and has been used in several community-based epidemiology studies [19], [21], [22]. In this instrument, 19 individual items generate 7 "component" scores: subjective sleep quality, sleep latency, sleep duration, habitual sleep efficiency, sleep disturbances, use of sleeping medication, and daytime dysfunction. A global score of subjective sleep quality (range 0–21) is then determined by the sum of the 7 component scores with the higher scores representing poorer subjective sleep quality [25], [26]. Habitual sleep efficiency was derived from the formula: hours of sleep/(get-up time - usual bedtime) ×100%. Sleep disturbance was derived from CPSQI items of “wake up in the middle of the night or early morning”, “have to get up to use the bathroom”, “cannot breathe comfortably”, “cough or snore loudly”, “feel too cold”, “feel too hot”, “had bad dreams”, and “have pain”. Higher scores indicate more sleep disturbances. Daytime dysfunction was derived from CPSQI items of “during the past month, how often have you taken medicine (proscribed or over the counter) to help your sleep” and “during the past month, how often have you had trouble staying awake while driving, eating meals, or engaging in social activity”. Higher scores indicate more daytime dysfunction. The respondents were diagnosed as poor sleepers if they obtained a CPSQI global score of >5 [27]. The criteria of poor sleeper yielded a sensitivity and specificity of 98% and 55%, respectively, in primary insomniacs *vs.* controls according to the diagnostic criteria for primary insomnia on the basis of the DSM-IV [27].

### Statistical analysis

The continuous variables were expressed as the mean and standard deviation (SD), and the categorical variables were expressed as frequencies (%). The Student t-test, analysis of variance (ANOVA), Pearson Chi-squared test and Cochran Mantel Haenszel Chi-squared were used to compare the continuous variables and categorical variables. The prevalence and 95% confidence intervals (CIs) of poor sleeper were calculated for the entire population, as well as by age and gender. A multiple logistic regression analysis was used to detect the risk factors associated with poor sleep quality. The models were adjusted for the demographic factors, and life styles (model 1) or combined with clinical and mental statuses listed in the relevant tables (model 2 and 3). In model 2, all of the clinical statuses that were significant in the univariate analysis were summed into one variable, the number of significant chronic illness, which included hypertension, osteoporosis, arthritis, migraine and heart disease. In model 3, each individual clinical disorder was entered into the model by itself.

All of the p-values and CIs were estimated in a two-tailed manner. Differences were considered to be statistically significant at p<0.05. The data were analyzed using SAS 9.3 (SAS Institute Inc., Cary, NC, USA).

## Results

### Characteristics of study participants

Among 1086 study participants, 622 (57.3%) were females. The average age of participants was 72.2 years (SD = 8.3), and they had an average of 11 years (SD = 4.5) of education. Nine percent and 7.8% of the participants were regular smoker and alcohol drinker. Among the participants, 9.8% were living alone, and 34.0% were doing exercise regularly. The most common chronic illnesses of the study participants were hypertension (57.9%), heart disease (33.4%), osteoporosis (23.4%), and arthritis (22.7%). The average scores of MMSE, CESD, and ZSAS were 27.3 (SD = 3.8), 8.0 (SD = 7.9), and 26.5 (SD = 6.4), respectively (Table 1).

**Table 1:** Socio-demographic characteristics and clinical disorders between good sleeper and poor sleeper.

	Total (n = 1086)	Poor sleeper (n = 451)	Good sleeper (n = 635)	p value
Female, n(%)	622 (57.3%)	285 (63.2)	337 (53.1)	0.001
Age, mean (SD)	72.2(8.3)	73.9 (8.4)	70.9 (8.1)	<0.001
BMI, mean (SD)	24.7(3.4)	24.6 (3.3)	24.8 (3.5)	0.561
Education duration, mean (SD)	11.0(4.5)	10.2 (4.8)	11.5 (4.3)	<0.001
Living single, n(%)	106 (9.8%)	65 (14.4)	41 (6.5)	<0.001
Regular smoker, n(%)	96 (8.9%)	36 (8.0)	60 (9.5)	0.393
Regular alcohol drinker, n(%)	84 (7.8%)	41 (9.1)	43 (6.8)	0.159
Doing exercise regularly, n(%)	366 (34.0%)	130 (29.0)	236 (37.5)	0.003
Number of chronic diseases, mean (SD)	1.4 (1.1)	1.7(1.1)	1.2(1.0)	<0.001
Hypertension, n(%)	629 (57.9%)	281 (62.3)	348 (54.8)	0.014
Diabetes, n(%)	177 (16.3%)	73 (16.2)	104 (16.4)	0.933
Osteoporosis, n(%)	254 (23.4%)	133 (29.5)	121 (19.1)	<0.001
Arthritis, n(%)	247 (22.7%)	136 (30.2)	111 (17.5)	<0.001
Chronic bronchitis, n(%)	125 (11.5%)	56 (12.4)	69 (10.9)	0.430
Migraine, n(%)	42 (3.9%)	23 (5.1)	19 (3.0)	0.076
Heart disease, n(%)	363 (33.4%)	179 (39.7)	184 (29.0)	<0.001
MMSE, mean (SD)	27.3 (3.8)	26.7 (4.4)	27.8 (3.3)	<0.001
CESD, mean (SD)	8.0 (7.9)	10.6 (8.9)	6.1 (6.6)	<0.001
ZSAS, mean (SD)	26.5 (6.4)	29.0 (7.1)	24.8 (5.2)	<0.001

Notes: BMI = Body Mess Index; MMSE = Mini-mental State Examination; CESD =  Center for Epidemiologic Studies Depression Scale; ZSAS =  Zung Self-Rating Anxiety Scale.

### Socio-demographic characteristics and clinical disorders between good sleepers and poor sleepers

The lifestyle characteristics and clinical disorders of the present sample are listed in Table 1. Poor sleepers had a higher proportion of females (63.2% *vs.*53.1%, p = 0.001), older subjects (73.9 *vs.* 70.9 years old, p<0.001) and a shorter duration of education (10.2 *vs.*11.5 years, p<0.001), comparing with good sleepers. More poor sleepers lived alone (14.4% *vs.* 6.5%, p<0.001), and less poor sleepers were currently engaged in physical exercise (29.0% *vs.* 37.5%, p = 0.003).

Comparing with good sleepers, poor sleepers demonstrated more hypertension (62.3% *vs.* 54.8%, p = 0.014), osteoporosis (29.5% *vs.* 19.1%, p<0.001), arthritis (30.2% *vs.* 17.5%, p<0.001) and heart disease (39.7% *vs.* 29.0%, p<0.001). Poor sleepers also had a lower MMSE score (26.7±4.4 *vs.* 27.8±3.3, p<0.001), a higher CESD score (10.6±8.9 *vs.* 6.1±6.6, p<0.001), and a higher ZSAS (29.0±7.1 *vs.* 24.8±5.2, p<0.001).

### Components of sleep quality

The participants, on average, went to bed at 10 pm, and rose in the morning at 6 am. Their average bedtime was 8.4 hours (SD = 1.3), and average night sleep duration was 6.9 hours (SD = 1.3). Seven components of sleep quality in the present sample population are listed in Table 2. The average sleep latency of the participants was 24.1 minutes (SD =  22.0), and 12.6% of the participants could not fall asleep within 30 minutes. In this sample population, 447 (41.2%) reported having less than 7 hours of sleep per night, 585 (53.9%) had a high habitual sleep efficiency of over 85%, and 185 (17.0%) used sleep medication within the past month at the time of interview. The mean scores for subjective sleep quality, sleep disturbances and daytime dysfunction were 2.0 (SD  =  0.8), 0.5 (SD  =  0.3), and 0.1 (SD  =  0. 4), respectively (Table 2).

**Table 2:** Gender and age specific scores of sleep quality components measured by the Chinese version of the Pittsburgh Sleep Quality Index.

			Gender			Age group			
		Total n = 1086	Male n = 464	Female n = 622	p value	60–69 n = 455	70–79 n = 412	> = 80 n = 219	P value
Subjective sleep quality☆, M (SD)		2.0(0.8)	2.0(0.7)	2.1(0.8)	0.002	2.0(0.8)	2.1(0.8)	2.1(0.8)	0.07
Sleep latency (min), M (SD)		24.1(22.0)	21.7(20.2)	25.8(23.1)	0.002	21.4(19.0)	25.5(22.7)	27.0(25.6)	0.002
Sleep latency, n (%)	<15 min	571 (52.6)	275 (59.3)	296 (47.6)	0.002	258 (56.7)	206 (50.0)	107 (48.9)	0.005
	16- min	379 (34.9)	142 (30.6)	237 (38.1)		158 (34.7)	147 (35.7)	74 (33.8)	
	30- min	105 (9.7)	36 (7.8)	69 (11.1)		32 (7.0)	43 (10.4)	30 (13.7)	
	>60 min	31 (2.9)	11 (2.4)	20 (3.2)		7 (1.5)	16 (3.9)	8 (3.7)	
Sleep duration (hour), M (SD)		6.9(1.3)	6.9(1.3)	6.8(1.3)	0.531	7.0(1.1)	6.7(1.3)	6.9(1.5)	0.013
Sleep duration, n (%)	<6 h	154 (14.2)	59 (12.7)	95 (15.3)	0.476	51 (11.2)	65 (15.8)	38 (17.4)	0.008
	6–7 h	293 (27.0)	132 (28.4)	161 (25.9)		111 (24.4)	125 (30.3)	57 (26.0)	
	7–8 h	551 (50.7)	232 (50.0)	319 (51.3)		257 (56.5)	196 (47.6)	98 (44.7)	
	>8 h	88 (8.1)	41 (8.8)	47 (7.6)		36 (7.9)	26 (6.3)	26 (11.9)	
Habitual sleep efficiency*, M (SD)		0.8(0.1)	0.8(0.1)	0.8(0.1)	0.056	0.9(0.1)	0.8(0.2)	0.8(0.2)	<0.001
Habitual sleep efficiency, n (%)	>85%	585 (53.9)	265 (57.1)	320 (51.4)	0.303	297 (65.3)	202 (49.0)	86 (39.3)	<0.001
	75–84%	231 (21.3)	92 (19.8)	139 (22.3)		96 (21.1)	91 (22.1)	44 (20.1)	
	65–74%	119 (11.0)	49 (10.6)	70 (11.3)		32 (7.0)	53 (12.9)	34 (15.5)	
	<65%	151 (13.9)	58 (12.5)	93 (15.0)		30 (6.6)	66 (16.0)	55 (25.1)	
Sleep disturbances★, M (SD)		0.5(0.3)	0.5(0.3)	0.5(0.3)	0.916	0.6(0.3)	0.7(0.3)	0.7(0.3)	<0.001
Use of sleeping medication, n (%)	Never	901 (83.0)	400 (86.2)	501 (80.5)	0.014	408 (89.7)	337 (81.8)	156 (71.2)	<0.001
	Yes	185 (17.0)	64 (13.8)	121 (19.5)		47 (10.3)	75 (18.2)	63 (28.8)	
Daytime dysfunction▾, M(SD)		0.1(0.4)	0.1(0.3)	0.1(0.4)	0.341	0.1(0.4)	0.1(0.4)	0.1(0.4)	0.971

Mean differences analyzed with t tests or ANOVA; proportional differences analyzed with Pearson Chi-squared test or Cochran-Mantel-Haenszel Chi-squared test.

M, mean; SD, standard deviation.

☆Scores range from 0 to 3; higher scores indicate poor subjective sleep quality.

* Habitual sleep efficiency was derived from the formula: hours of sleep/(get-up time - usual bedtime) *100%.

★Derived from Pittsburgh Sleep Quality Index (PSQI) items 5b–5j; scores range from 0 to 3; higher scores indicate more sleep disturbances.

▾Derived from PSQI items 7–8; scores range from 0 to 3; higher scores indicate more daytime dysfunction.

Females scored significantly poorer on sleep latency (25.8 min *vs.* 21.7 min, p  = 0.002) and subjective sleep quality (2.1 *vs.* 2.0, p  = 0.002). Males were more reluctant to obtain sleep medication treatment compared to females (13.8% *vs.* 19.5%, p = 0.014). No significant differences were found between males and females in sleep duration, habitual sleep efficiency, sleep disturbances and daytime dysfunction. Older people had longer sleep latency, poorer sleep efficiency, more sleep disturbance and poorer subjective sleep quality. More people with older age used sleeping medication than younger counterpart (Table 2).

### Prevalence of poor sleep quality

The CPSQI global score in females was 5.9 (95%CI = 5.6–6.2), which was significantly higher than that in males (5.2, 95% CI = 4.9–5.5, p<0.001). It was increased by enhanced age, from 4.8 (95% CI = 4.5–5.1) in people aged 60–69 years to 6.6 (95%CI = 6.1–7.2) in people aged ≥80 years (p value for trend<0.001). In this study, 41.5% (95% CI = 38.6–44.5%) of the participants were assessed as poor sleeper by global CPSQI score less than 5. The prevalence of poor sleeper in females was 45.8% (95% CI = 41.9–49.7%), which was significantly higher than that in males (35.8%, 95% CI = 31.4–40.1%). The prevalence of poor sleep quality increased with enhanced age, from 32.1% (95% CI = 27.8–36.4%) in people aged 60–69 years to 52.5% (95% CI = 45.9–59.1%) in people aged ≥80 years (p value for trend<0.001) (Table 3).

**Table 3:** The Chinese version of the Pittsburgh Sleep Quality Index global score and prevalence of poor sleep quality of participants by gender and age groups.

		N	CPSQI global score mean(95%CI)	p value	Poor sleep quality* prevalence (95%CI)	p value
Total		1086	5.6(5.4–5.8)		41.5(38.6–44.5)	
Gender	Male	464	5.2(4.9–5.5)	<0.001	35.8(31.4–40.1)	<0.001
	Female	622	5.9(5.6–6.2)		45.8(41.9–49.7)	
Age group	60-	455	4.8(4.5–5.1)	<0.001▽	32.1(27.8–36.4)	<0.001▽
	70-	412	5.9(5.6–6.3)		46.1(41.3–50.9)	
	80-	219	6.6(6.1–7.2)		52.5(45.9–59.1)	

* Chinese version of the Pittsburgh Sleep Quality Index global score < = 5.

▽p value for trend.

### Risk factors associated with poor sleep quality

The multiple logistic regression analysis of socio-demographic characteristics and clinical disorders on sleep quality are presented in Table 4. Females had a higher risk for poor sleep quality after adjusting for socio-demographic characteristics in model 1 (OR = 1.34 [95% CI =  1.03–1.75]), p = 0.031); however, this association was not significant after being adjusted for clinical disorders in model 2 (OR = 1.09 [95% CI = 0.81–1.45], p = 0.573). After adjusting for other covariates in model 2, age was positively associated with sleep quality with an OR = 1.03 (95% CI = 1.01–1.05, p = 0.004). The duration of education was inversely associated with a risk of poor sleep quality (OR = 1.05 [95% CI = 1.01–1.09], p = 0.011). Those who lived alone had a higher prevalence of poor sleep quality (OR = 1.62 [95% CI = 1.02–2.57], p = 0.041). Subjects with a higher ZSAS score (OR = 1.09(95% CI = 1.05–1.13), p<0.001) were positively associated with sleep quality. Subjects with more chronic disease suffered more from sleep disturbance (OR = 1.18 [95% CI =  1.03–1.36, p = 0.014). Model 3 showed that each individual clinical disorder was positively associated with sleep quality, however, only arthritis showed a significantly positive association with sleep quality (OR = 1.45 [95% CI = 1.05–2.01], p = 0.025).

**Table 4:** Multiple logistic regression models of factors associated with poor sleep quality.

	model 1	p value	model 2	p value	model 3	p value
Female gender	1.34(1.03–1.75)	0.031	1.09(0.81–1.45)	0.573	1.07(0.80–1.44)	0.634
Age	1.04(1.02–1.05)	<0.001	1.03(1.01–1.05)	0.004	1.03(1.01–1.05)	0.003
Education duration	1.04(1.02–1.08)	0.008	1.05(1.01–1.09)	0.011	1.04(1.01–1.08)	0.014
Living single	1.95(1.27–2.99)	0.002	1.62(1.02–2.57)	0.041	1.62(1.02–2.58)	0.040
Doing exercise regularly	0.67(0.51–0.87)	0.003	0.75(0.56–1.00)	0.051	0.76(0.57–1.01)	0.057
MMSE	–	–	0.99(0.95–1.04)	0.749	0.99(0.95–1.04)	0.737
CESD	–	–	1.02(1.00–1.05)	0.113	1.02(0.995–1.05)	0.107
ZSAS	–	–	1.09(1.05–1.13)	<0.001	1.09(1.05–1.12)	<0.001
Number of chronic disease	–	–	1.18 (1.03–1.36)	0.014	–	–
Hypertension	–	–	–	–	1.08(0.82–1.43)	0.586
Osteoporosis	–	–	–	–	1.11(0.80–1.55)	0.529
Arthritis	–	–	–	–	1.45(1.05–2.01)	0.025
Migraine	–	–	–	–	1.37(0.67–2.81)	0.387
Heart disease	–	–	–	–	1.13(0.84–1.51)	0.419

*Notes:* Models were adjusted for demographic factors and life styles (model 1) or combined with potential clinical status listed in the relevant tables (model 2 & 3). In model 2, all the clinical illnesses significant in the univariate analysis were summed into one variable-the number of significant chronic illness. In model 3, each individual clinic disorder was entered into the model by themselves.

## Discussion

The current population-based study used the validated CPSQI to evaluate the sleep quality and its risk factors in a sampled population with elderly in urban China. The overall prevalence of poor sleep quality (defined as CPSQI >5) was 41.5% (95% CI = 38.6–44.5%) which is comparable with that in western populations [1] and Hongkong [22]. Aging, living alone, low education, anxiety and the number of chronic disease were found to be significantly associated with poor sleep quality.

In the recent 10 years, there have been several population-based studies conducted in China to explore the sleep problems in Chinese elderly. The first study was conducted in 1034 elderly aged ≥70 years in Hongkong in late 1990's. This two-phase study used a sleep symptom questionnaire in phase 1, and made the insomnia diagnosis according to DSM-IV by psychiatrists in phase 2. Occasional or persistent sleep disturbance were reported in 75%, and insomnia were diagnosed in 38.2% of the study sample [17]. A study in Taiwan used a symptom questionnaire and DSM-IV criteria, and demonstrated the prevalence of one-month insomnia of 6% [19]. In mainland China, some studies used a structured sleep questionnaire focusing on 3 insomnia symptoms: difficulty initiating sleep (DIS), difficulty maintaining sleep (DMS), and early morning awakening (EMA) [18], [20]. Xiang YT et al reported that, among 1159 individuals aged ≥65 years in Beijing and Shanghai, 14% suffered at least one of the 3 insomnia symptoms [15]. Liu X et al surveyed 1679 individuals aged ≥65 years in 5 cities of Shandong province. The prevalence of insomnia symptoms (defined as at least one of the 3 insomnia symptoms) was reported as 32.9%. In that study, questions were also designed to ask for the daytime consequences due to insomnia, eg. mood disturbance, irritability, fatigue, daytime sleepiness, poor concentration, and headache. The prevalence of insomnia disorder (defined as at least one of the 3 insomnia symptoms with one or more consequent symptoms) was reported as 8.9% [18].

Sleep quality in most studies was simply measured in levels of “well, common, or poor” by one question. A cross-sectional study surveyed 3289 subjects aged 50 to 70 years in Beijing and Shanghai, and 16.2% subjects reported poor sleep quality [24]. In the 2005 wave of the Chinese Longitudinal Healthy Longevity Survey, 35% of 15638 individuals aged ≥65 years reported the “fair to very bad” sleep quality [23]. The Study on Global Ageing and Adults Health demonstrated that, 15.7% of 7154 Chinese individuals aged ≥60 years reports moderate to severe sleep complains [16]. These results, however, were not comparable due to the difference of the outcome definition and measurement questions, besides the detection procedure and demographic characteristics of study sample.

The CPSQI has been validated to be a sensitive, reliable, and valid outcome assessment tool for use in population-based studies of primary insomnia. It assesses the quantitative aspects of sleep, such as subjective sleep quality, sleep latency, sleep duration, habitual sleep efficiency, sleep disturbances, use of sleeping medication, and daytime dysfunction [27]. It also considered the physical and cognitive symptoms of poor sleep quality include tiredness, loss of concentration, low pain threshold, anxiety, nervousness, irrational thoughts, hallucinations, loss of appetite, constipation, and being accident-prone [34]. It can sufficiently evaluate the complete concept of sleep quality in an epidemiological study by using a quantitative measurement. To our knowledge, only 2 published studies used CPSQI in Chinese population. A study in Hongkong reported a 47.1% of the insomnia (defined by CPSQI global score >5) prevalence among people aged ≥60 years. However, that survey was conducted via a telephone interview with a low response rate of 47% [22]. Recently, a study with a face-to-face interview using CPSQI was conducted in rural area of Anhui province. It was reported that 49.7% of the 2416 participants aged ≥60 years slept poorly. However, the poor sleep quality in that study was defined as a CPSQI global score of >7 [21]. Our study enriches the understanding of the prevalence of sleep quality in Chinese elderly by providing data from an urban community, a face-to-face interview, and a completely analysis of CPSQI scores. It is reasonable to believe the prevalence of poor sleep quality in the current urban Shanghai population is less than those in rural Anhui province. The lower prevalence rate may due to high education level and better health status, although other confounding factors like age structure can not be eliminated.

There were some studies in China with large sample size, just to survey the sleep duration among the elderly. In the Shanghai Women's Health Study, 68,832 women aged from 40 to 70 years reported their average sleep duration of 7 hours [35]. The average sleep duration was 6.9 hours in 28,670 Chinese elderly Chinese aged 50 to 85 years in the Guangzhou Biobank Cohort Study [36]. The average sleep duration (6.9 hours) obtained in our study was very similar to that from those two studies.

Consistent with existing epidemiological data, our findings showed that poor sleep quality generally increased with age and was more prevalent in females [37]. A shorter education duration and living alone were positively associated with poor sleep quality. Regularly performing physical exercise was a protective factor of sleep quality [20], [22], [38], [39]. However, after adjusting for potential confounders, the association between gender and poor sleep quality was not statistically significant. In the present study, females had less education (9.9 *vs.* 12.3 years, p<0.001) and a higher proportion of females lived alone (13.7% *vs.* 4.5%, p<0.001) than that of males. More females also suffered from depression, anxiety and heart disease. These findings, consistent with a study in Hongkong [22], suggested that socioeconomic factors and chronic disease, rather than gender, may contributed to the significant association observed between the female elderly and poor sleep quality.

Consistent with previous reports [17], [19], [40], depression and anxiety were associated with sleep quality in the present Chinese sample population. The reason of non-significant association between depression and sleep quality in model 2 might be the high correlation between depression and anxiety (correlation coefficient = 0.715, p<0.001). Medical conditions like obstructive airway diseases, especially concurrent asthma and chronic bronchitis arthritis, heart diseases or a painful physical affliction (e.g. back pain) had been found to be associated with sleep disorder [19], [41], [42]. In the present study, the significant association between the number of chronic disorders, rather than the individual clinical disorders and sleep quality, provided new insights into the association between the presences of multiple comorbidities and sleep disturbance. The impact of each individual chronic illness on sleep disturbance might not be significant enough. There might be some kind of synergy effect of pain, physical and psychiatric disorders on poor sleep quality. Our findings were consistent with previous studies. Mellinger et al observed that the prevalence of insomnia symptoms was higher in subjects with two or more health problems [43]. Hayashino et al found out that the number of comorbid conditions was associated with sleep quality after adjustment of depression [34]. Chiu HF, et al and Xiang YT, et al also indicated that sleep disorder was associated with ‘major medical conditions’ in Chinese population [17], [20].

The strength of this study is that the data are reliable and well-controlled; the data were obtained from face-to-face interviews by trained neurologists and qualified psychiatrists rather than via a telephone interview. In addition, the cases were determined using a validated CPSQI rather than unstructured questions. All of the clinical disorders were obtained and verified from a medical record rather than from self-reported simple questions.

Nonetheless, there are several limitations in this study. First, the CPSQI defines a criteria of poor sleepers yielding a sensitivity and specificity of 98% and 55%, respectively, in primary insomniacs *vs.* controls according to the diagnostic criteria for primary insomnia on the basis of the DSM-IV [27]. Low specificity means the risk of normal subjects be misclassified as poor sleepers. Thus, the prevalence of poor sleepers in our study might be overestimated. However, CPSQI is still a validated measurement for sleep quality and better than those unstructured questions. Second, the use of cross-sectional data prohibits any assessment of the cause-and-effect relationship between sleep disturbances and physical and psychiatric disorders. The cause-and effect relationship may be verified in the later follow-up study for this cohort. Third, our study population is less representative, since the population in Shanghai has a higher proportion of elderly, higher proportion of empty-nesters who live alone and a higher distribution of education than that in the general population in China. Their living condition in urban is better than rural areas. Because of complex biological and psychosocial determinants, it is hard to tell whether urban residents will suffer more on poor sleep quality or not. Our study provided data in the most developed urban area in mainland China. However, these results can hardly be generalized to other Asian countries, or even to other countries in the world.

Our study suggests that approximately 2 out of 5 elderly people living in urban China slept poorly and the prevalence rate increased rapidly by aging. The application of the study results may be more relevant in public health than clinical practice. Growing attention and comprehensive countermeasures involving psycho-social and personal activities might alleviate the sleep problem in the elderly. Furthermore, population-based studies with a larger sample size and a longitudinal follow-up may help to determine the related factors and potential intervention of sleep quality.
